# Social Cognition in young onset Alzheimer’s Disease: what pattern of impairment compared to late‐onset disease?

**DOI:** 10.1002/alz.095344

**Published:** 2025-01-09

**Authors:** Tatiana Belfort, Marcela Moreira Lima Nogueira, Isabel Lacerda, Maria Alice Baptista, Julia Gaigher, Rogeria Rangel, Natalie Souza, Marcia C. N. Dourado

**Affiliations:** ^1^ Federal University of Rio de Janeiro, Rio de Janeiro Brazil; ^2^ Federal University of Rio de Janeiro, Rio de Janeiro, Rio de Janeiro Brazil

## Abstract

**Background:**

People with young‐onset Alzheimer`s disease (YOAD) is a diagnosis given when the neurocognitive process begins before the age of 65 and often presents more global impairments, and the course of the disease is faster. In contrast, in late‐onset (LOAD), the most prominent loss occurs in short‐term memory. Therefore, the age at onset of the disease may affect global functioning in different ways. The present study aims to investigate the relationship between SC, global cognition, and other clinical variables in young and late‐onset people with AD and their caregivers.

**Method:**

Using a cross‐sectional design, we included 48 people with YOAD and 118 with LOAD, and their caregivers. We assessed social cognition, global cognition, quality of life, dementia severity, mood, functionality, neuropsychiatric symptoms, and caregiver burden.

**Result:**

The YOAD group was more impaired in general cognition, as well MMSE scores (P = 0.018, d = 0.41) as Adas cog (P = 0.002, d = 0.06), had a worse quality of life (QoL‐AD) (P = 0.036, d = 0.36) and more neuropsychiatric symptoms (NPI) (P = 0.044, d = 0.35). However, Social Cognition presented a stable pattern of impairments in YOAD, which do not follow the global deficits. The multifactorial regression analyses showed that in both groups the functionality was related to Social Cognition, YOAD (P = 0.035), and LOAD (P = 0.001).

**Conclusion:**

Our study findings support that in young‐onset Alzheimer’s disease, even a more global impairment than compared to late‐onset, the social cognition remains established.

